# *De novo* neurogenesis by targeted expression of *atoh7* to Müller glia cells

**DOI:** 10.1242/dev.135905

**Published:** 2016-06-01

**Authors:** Katharina Lust, Rebecca Sinn, Alicia Pérez Saturnino, Lázaro Centanin, Joachim Wittbrodt

**Affiliations:** 1 Centre for Organismal Studies (COS) Heidelberg, Im Neuenheimer Feld 230, Heidelberg 69120, Germany; 2 The Hartmut Hoffmann-Berling International Graduate School of Molecular and Cellular Biology (HBIGS), Heidelberg University, Heidelberg, Germany

**Keywords:** Müller glia, Atoh7, Medaka, Retina, LexPR system, Lineage tracing

## Abstract

Regenerative responses in the vertebrate CNS depend on quiescent radial glia stem cells, which re-enter the cell cycle and eventually differentiate into neurons. The entry into the cell cycle and the differentiation into neurons are events of opposite nature, and therefore efforts to force quiescent radial glia into neurons require different factors. Here, we use fish to show that a single neurogenic factor, Atoh7, directs retinal radial glia (Müller glia, MG) into proliferation. The resulting neurogenic clusters differentiate *in vivo* into various retinal neurons. We use signaling reporters to demonstrate that the Atoh7-induced regeneration-like response of MG cells is mimicked by Notch, resembling the behavior of early progenitors during retinogenesis. Activation of Notch signaling in MG cells is sufficient to trigger proliferation and differentiation. Our results uncover a new role for Atoh7 as a universal neurogenic factor, and illustrate how signaling modules are re-employed in diverse contexts to trigger different biological responses.

## Abstract (precis)

**Highlighted article:** Induced activation of *atoh7* in Müller glia cells *in vivo* is sufficient to drive cell cycle re-entry and proliferation, followed by the formation of neurogenic clusters and *de novo* neurogenesis.

## INTRODUCTION

Most adult animals show some degree of ability to regenerate lost cell types, tissues and even organs (Aguirre et al., 2013; Birnbaum and Sánchez Alvarado, 2008; Blanpain and Fuchs, 2014; Lamba et al., 2008; Miyajima et al., 2014; Tanaka and Reddien, 2011). The regeneration potential is very variable among different organisms and decreases with growing complexity. Planarians show the highest degree of regeneration and plasticity and are able to regenerate their entire body from a single neoblast (Wagner et al., 2011). Among vertebrates, fish show a high regenerative capacity and can regenerate fins, the heart, neurons and various other organs (Birnbaum and Sánchez Alvarado, 2008; Blanpain and Fuchs, 2014; Gemberling et al., 2013; González-Rosa et al., 2012; Kaslin et al., 2008; Kikuchi and Poss, 2012; Knopf et al., 2011; Lamba et al., 2008; Singh et al., 2012). Regeneration often involves quiescent stem cells that are re-activated upon injuries or other major challenges (Blanpain and Fuchs, 2014; Gemberling et al., 2013). The identification of signaling pathways and, ideally, individual factors responsible for the switch from quiescence to activity is of exceptional interest as an entry point for regenerative therapies.

Müller glia (MG) cells are considered the radial glia of the vertebrate retina (Bernardos et al., 2007; Raymond et al., 2006), which in addition contains differentiated neurons as cone and rod photoreceptors, horizontal and amacrine cells, bipolar cells and retinal ganglion cells (Centanin and Wittbrodt, 2014; Dowling, 1987). MG cells represent a population of quiescent multipotent stem cells during post-embryonic life (Bernardos et al., 2007; Moshiri et al., 2004). Zebrafish and goldfish MG cells are reported to react to homeostatic signals by production of rod photoreceptor cells (Bernardos et al., 2007; Johns and Fernald, 1981). Additionally, MG cells mediate regeneration in the fish retina by re-entering the cell cycle (Goldman, 2014; Gorsuch and Hyde, 2014; Lamba et al., 2008; Lenkowski and Raymond, 2014) and re-establishing retinal cell types (Bernardos et al., 2007; Fausett and Goldman, 2006; Fimbel et al., 2007) to reconstitute the function of compromised neuronal networks (Mensinger and Powers, 2007). The regenerative capacity of the MG cell population has been extensively studied by different injury models, such as laser ablation, neurotoxin treatment, surgical removal or puncture, localized heat and constant intense-light treatment (Bernardos et al., 2007; Fausett and Goldman, 2006; Fimbel et al., 2007; Gorsuch and Hyde, 2014; Kassen et al., 2007; Yurco and Cameron, 2005). However, only a few factors have been implicated functionally in retinal regeneration by the use of morpholinos (Ascl1a, TNFα), and *in vivo* transfection (Ascl1a) (Fausett and Goldman, 2006; Nelson et al., 2013).

The transcription factor Atoh7 is involved in many aspects of early neurogenesis in the vertebrate retina (Brown et al., 2001; Kay et al., 2001; Poggi et al., 2005). In fish, *atoh7* expression starts during the final divisions of retinal progenitor cells (RPCs), and it is necessary for the generation of retinal ganglion cells (RGCs) during retinogenesis. Mutants lacking *atoh7*, such as the *lakritz* mutant in zebrafish (Kay et al., 2001), lack RGCs but no other cell types of the neural retina. Conversely, overexpression of *atoh7* in RPCs leads to a preferential differentiation towards RGCs (Feng et al., 2010; Kanekar et al., 1997; Kay et al., 2001; Liu et al., 2001; Sinn et al., 2014; Wang and Harris, 2005). Although Atoh7 is only necessary to produce RGCs, Atoh7-positive RPC descendants also include photoreceptors, amacrine and horizontal cells (Kay et al., 2001; Ma et al., 2004). *atoh7* has also been shown to be upregulated in regeneration paradigms (Fimbel et al., 2007; Sherpa et al., 2008). However, its role in the process of regeneration could not be assessed owing to the lack of a conditional genetic system allowing its inducible and transient expression in MG cells.

In the present study, we find that *atoh7* is expressed in proliferating progenitors in the ciliary marginal zone (CMZ) as well as in proliferating MG cells and progenitors after retinal injury. To address the potential of Atoh7 in triggering cell cycle re-entry of quiescent MG cells of the medaka retina, we use the mifepristone-inducible Lex^PR^/*OP* transactivation system (Emelyanov and Parinov, 2008). We show that targeted expression of *atoh7* in MG cells is sufficient to drive them into the cell cycle. We also report that *atoh7* expression activates Notch signaling in a cell-specific manner, and inducible activation of Notch in MG cells recapitulates the mitotic effects of Atoh7. The re-activated MG cells form clonal neurogenic clusters and long-term lineage analysis demonstrates that they differentiate into retinal cell types. Our study identifies Atoh7 as sufficient to trigger a regeneration-like response in the absence of additional stimuli, activating proliferation and differentiation of individual quiescent MG cells *in vivo*.

## RESULTS

### *atoh7* is expressed in proliferating progenitors of the post-embryonic CMZ and in MG cells after injury

**Fig. 1.**
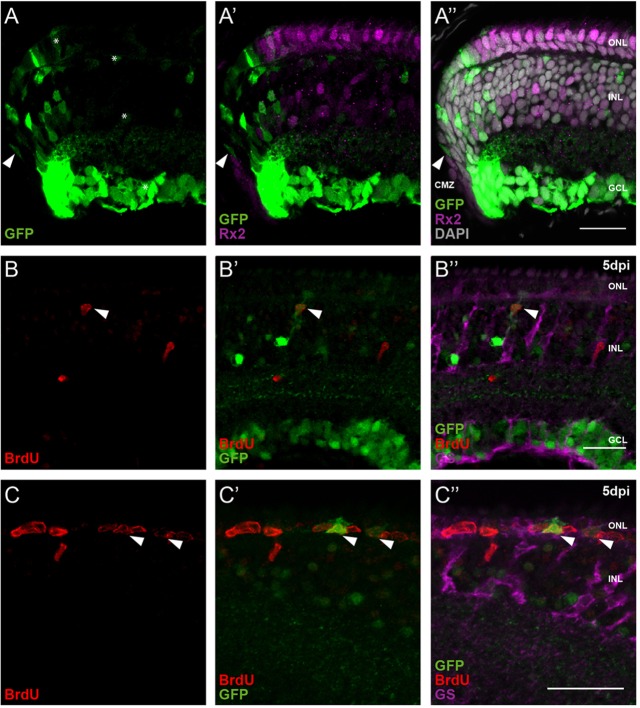
**Atoh7 marks proliferating progenitors in the CMZ and the central retina after injury.** (A-A″) *atoh7*-driven EGFP (green) expression (arrowheads) is detected in cells directly adjacent to the Rx2 (magenta) expression domain and in cells that are exiting the transient amplifying zone. The EGFP is still retained by differentiated retinal ganglion cells, amacrine cells, horizontal cells and photoreceptor cells (asterisks). (B-C″) Needle injuries of the retina induce *atoh7*-driven EGFP (green) expression in proliferating, BrdU-positive (red) MG cells in the INL, which are labeled by GS (magenta) (B-B″, arrowheads). Additionally, proliferating, BrdU-positive cells in the ONL also express EGFP after injuries (C-C″, arrowheads). *n*=12 fish, data obtained from three independent experiments. CMZ, ciliary marginal zone; ONL, outer nuclear layer; INL, inner nuclear layer; GCL, ganglion cell layer. Scale bars: 20 µm.

To investigate the role of Atoh7 during retinal growth and regeneration, we performed an expression analysis using an *atoh7* transcriptional reporter (*atoh7*::EGFP), which gives a direct readout of *atoh7* transcriptional activity (Del Bene et al., 2007). In the post-embryonic retina of medaka, we detected EGFP in RGCs, amacrine cells, horizontal and photoreceptor cells close to the CMZ (Fig. 1A). This expression indicates that Atoh7-positive progenitors derived from the CMZ give rise to these cell types, reminiscent of the situation during retina development (Poggi et al., 2005).

During retinal development, *atoh7* expression is confined to differentiating RPCs. Interestingly, our analysis uncovered a novel expression domain of *atoh7* in the peripheral CMZ. We found transient expression in progenitors exiting the stem cell niche, directly adjacent to the expression of *retinal homeobox gene two* (*rx2*) (Fig. 1A′,A″), which marks retinal stem cells (Reinhardt et al., 2015). As expected for proliferating progenitors, Atoh7-positive cells in the post-embryonic CMZ incorporate the thymidine analog bromodeoxyuridine (BrdU) when applied in a short pulse (16 h) (Fig. S1A-A‴). The expression of *atoh7* in the CMZ close to retinal stem cells suggests a role in proliferating, uncommitted progenitors.

In medaka hatchlings, MG cells do not display proliferation in the absence of injury (Fig. S1B-B‴). To investigate whether *atoh7* expression is upregulated in cells responding to retinal injury by proliferation, we performed needle injuries, placed the fish in BrdU for up to 5 days and analyzed the expression of the *atoh7* reporter in BrdU-positive cells of the central retina at time points starting at 1 day post injury (dpi). As in the CMZ, we found at 4 and 5 dpi a small number of EGFP-positive, BrdU-positive cells that were also positive for the MG marker glutamine synthetase (GS), consistent with the transient activity of *atoh7* in proliferating progenitors. We detect GFP-positive, BrdU-positive cells both in the inner nuclear layer (INL), representing MG cells (Fig. 1B-B″), as well as in the outer nuclear layer (ONL), representing MG cells transiting to progenitor cells that have responded to the injury by interkinetic migration of their nuclei towards the photoreceptor layer (Fig. 1C-C″).

These results argue for an early role of Atoh7 in the proliferation of retinal progenitors during retinal homeostasis and regeneration.

### An inducible system to activate gene expression in MG cells

**Fig. 2.**
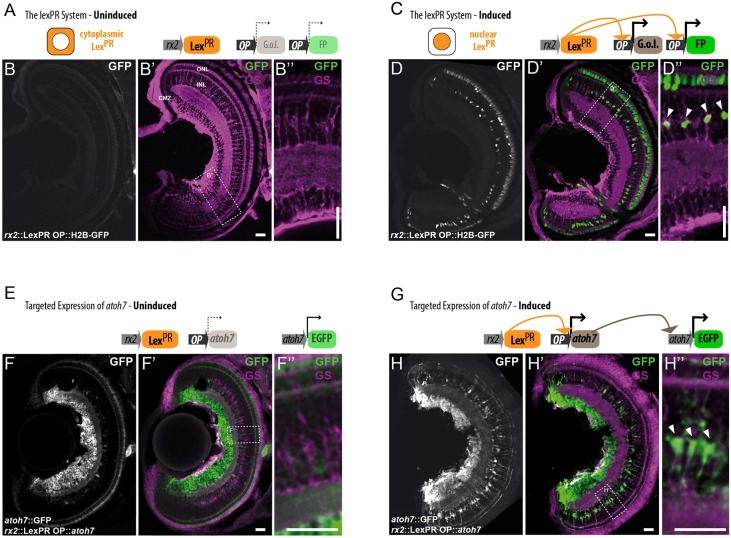
**The Lex^PR^ system is suitable** **for targeting** **gene expression to MG cells.** (A-D″) The LexPR system allows targeted and inducible gene expression in medaka. In the uninduced state, the Lex^PR^ transactivator is retained in the cytoplasm, *OP* elements are inactive and genes of interest (G.o.I.) and fluorophores (FPs) are not expressed (A-B″). Upon induction, Lex^PR^ translocates into the nucleus and activates G.o.I. and FPs (C-D″). GFP expression (white/green) is only detected in induced fish in all different *rx2* expression domains: the CMZ, the INL and the ONL. GFP-positive cells in the INL are also GS-positive (magenta) (D-D″, arrowheads). (E-H″) Targeted expression of *atoh7* results in a transcriptionally active factor. *atoh7*::EGFP expression (white/green) is confined to RGCs in the central uninduced retina (F-F″). Upon induction, the targeted Atoh7 can activate its own promoter in GS-positive MG cells (magenta) leading to GFP expression (white/green) (G-H″, arrowheads). Scale bars: 20 µm.

To address the role of Atoh7 in proliferation of MG cells, we used the Lex^PR^ inducible system (Emelyanov and Parinov, 2008) to trigger *atoh7* expression in MG cells of the differentiated medaka retina. The Lex^PR^ system relies on the expression of a trans-activating element (Lex^PR^), which only upon addition of mifepristone binds to one or more *operator-promoter* (*OP*) elements to drive gene expression (Emelyanov and Parinov, 2008) (see scheme in Fig. 2A,C). To drive expression of the Lex^PR^ to differentiated MG cells, we used the cis-regulatory element of *rx2* (Martinez-Morales et al., 2009), which also targets photoreceptor cells and retinal stem cells in the CMZ (Inoue and Wittbrodt, 2011; Reinhardt et al., 2015).

The activity of the *rx2*::Lex^PR^ transgenic line was monitored by combining it with an *OP*-driven fluorescent protein (*OP*::EGFP or *OP*::H2B-EGFP) (Fig. S2A-B″). In the absence of the drug, no reporter expression was detected in *rx2*::Lex^PR^ *OP*::EGFP fish. Mifepristone reliably triggered EGFP expression in MG cells and the other *rx2* expression domains of the mature medaka retina (Fig. 2A-D″; Fig. S2A-B″). We used this system to perform a targeted analysis of *atoh7* expression in differentiated MG cells.

### Induction of *atoh7* activity triggers MG cell proliferation

To address whether Atoh7 is sufficient to activate proliferation of quiescent MG cells, we targeted *atoh7* expression to MG cells by induction of an *rx2*::Lex^PR^ *OP*::*atoh7* in hatchling fish. Upon mifepristone treatment, we detected *atoh7* mRNA expression in MG cells, photoreceptors and the CMZ (Fig. S3B-B″). To address whether the conditionally expressed Atoh7 was transcriptionally active, we used an Atoh7 transcriptional reporter. Atoh7 was shown to activate its own regulatory sequence (Brown et al., 2001; Del Bene et al., 2007; Matter-Sadzinski et al., 2001; Skowronska-Krawczyk et al., 2004; Souren et al., 2009), such that the transgenic *atoh7*::EGFP line gives a direct readout of Atoh7 transcriptional activity (Del Bene et al., 2007) (Fig. 2E-F″). When we induced *atoh7* expression in the *atoh7*::EGFP line, EGFP was present in MG cells and in the CMZ (Fig. 2G-H″). However, no EGFP was detected in photoreceptors in the ONL, even though *atoh7* mRNA was detected there (Fig. S3B-B″). Our data indicate that *atoh7* transcription is induced in all *rx2* expression domains. However, transcriptionally active Atoh7 protein is only present in MG cells and the CMZ.

**Fig. 3.**
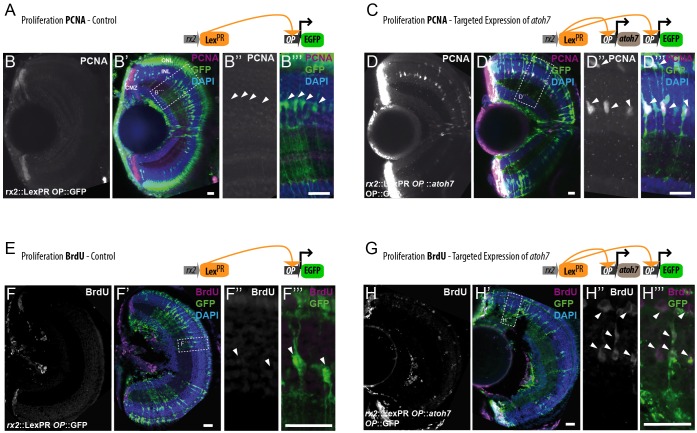
**Atoh7 induces MG cells to re-enter the cell cycle.** (A-D‴) Targeted expression of *atoh7* upregulates PCNA expression in MG cells. Upon induction, *rx2*-driven Lex^PR^ activates either EGFP expression in controls (A) or simultaneous *atoh7* and EGFP expression in experimental hatchlings (C). PCNA-positive cells (white/magenta) are detected in the transit amplifying zone of the CMZ (A), but not among MG cells (green) (B,B‴, arrowheads) in control retinae (*n*>8 fish, data obtained from two independent experiments). Targeted expression of *atoh7* results in PCNA upregulation in Rx2-positive cells of the INL and the ONL, colocalizing with EGFP expression (D,D′; arrowheads in D″,D‴) (*n*>8 fish, data obtained from two independent experiments). (E-H‴) Targeted expression of *atoh7* drives MG cells into S phase. BrdU incorporation was assessed in *rx2*::Lex^PR^ *OP*::EGFP controls (E) or *OP*::EGFP *OP*::*atoh7* experimental hatchlings (G). BrdU incorporation (white/magenta) was detected in the CMZ (F,F′) but not in MG cells (green) (F″,F‴, arrowheads) of control retinae (*n*>10 fish, data obtained from three independent experiments). Targeted *atoh7* expression results in BrdU incorporation (white/magenta) by EGFP-positive MG cells (green) in the central retina (H,H′), highly colocalizing with EGFP expression (H″,H‴, arrowheads) (*n*>10 fish, data obtained from three independent experiments). Scale bars: 20 µm.

To assess the effect of Atoh7 on MG cell proliferation, we analyzed the presence of mitotic cells within the central domain of induced *rx2*::Lex^PR^ *OP*::*atoh7 OP*::EGFP retinae. Proliferating cell nuclear antigen (PCNA) staining of retinae from control fish is restricted to peripheral progenitors located in the CMZ (Fig. 3A-B‴). Retinae from induced fish showed upregulation of PCNA at 2 days post induction in cells of the central retina, in addition to the CMZ domain (Fig. 3C,D). Based on the expression of EGFP and their morphology, we could identify that the mitotic cells corresponded to Atoh7-positive MG cells (Fig. 3D′-D‴). Additionally, we complemented this data with BrdU incorporation assays. Fish were induced for 4 days together with a BrdU pulse or kept as uninduced controls with a BrdU pulse lasting 4 days. Retinae from control fish showed a narrow domain of BrdU incorporation in the proliferative domain of the CMZ, but no incorporation in the central, differentiated INL (Fig. 3E-F‴). In induced *rx2*::Lex^PR^ *OP*::*atoh7* retinae, we found that the EGFP-positive MG cells incorporated BrdU with no central-peripheral preference (Fig. 3G-H‴), demonstrating that they re-enter the cell cycle and go through an S phase. These data demonstrate that the inducible expression of a single transcription factor, Atoh7, is sufficient to trigger proliferative activity *in vivo* in the otherwise quiescent MG cells of the fish retina.

### Induction of *atoh7* in MG cells activates Notch signaling

Notch signaling is a well-known regulator of neurogenesis in many different systems, including the developing fish neural retina (Baye and Link, 2008; Bernardos et al., 2005; Cayouette et al., 2006; Livesey and Cepko, 2001; Scheer et al., 2001). Perturbation of Notch activity in fish embryonic retinal progenitor cells results in proliferation/differentiation unbalance (Chiodini et al., 2013; Clark et al., 2012; Del Bene et al., 2008), and feedback loops involving *atoh7* and target genes of the Notch pathway were recently reported (Chiodini et al., 2013).

**Fig. 4.**
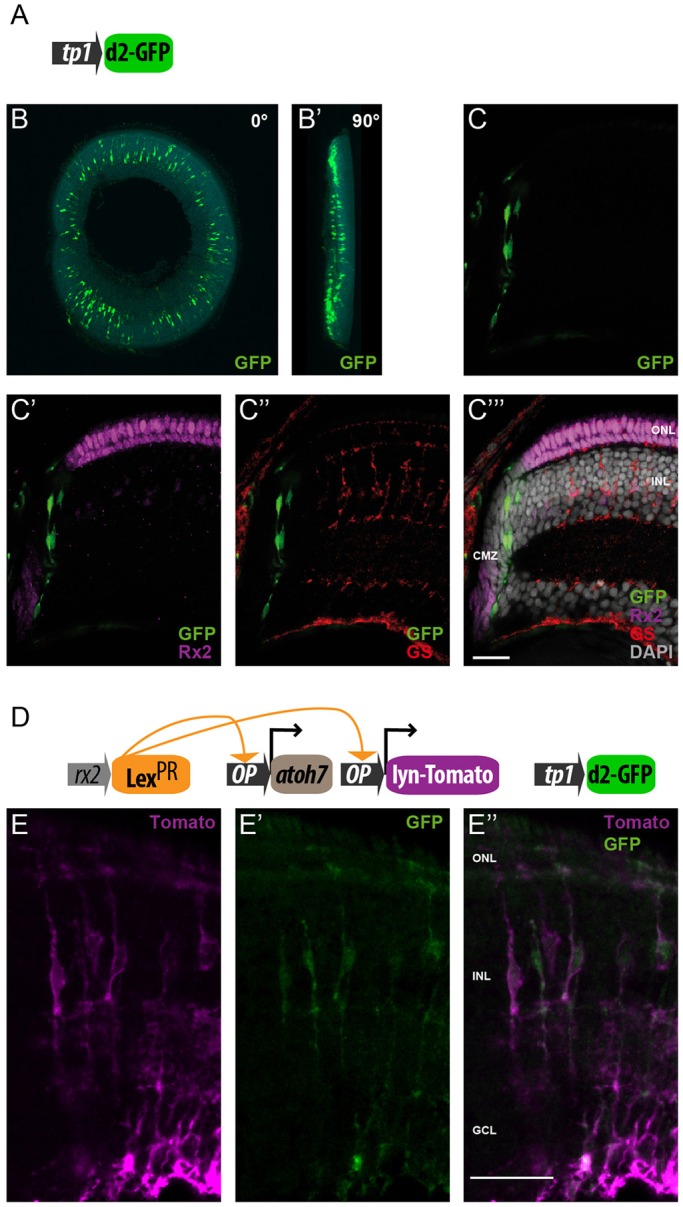
***atoh7* induction in MG cells activates Notch signaling.** (A-C‴) The *tp1-MmHbb*:d2GFP Notch transcriptional reporter (A) is activated in the peripheral retina of hatchling fish (B-C‴). GFP expression (green) is detected close to the CMZ but is non-overlapping with Rx2 (magenta) (C′). The reporter is not active in the central retina; no overlap with GS (red) can be detected (C″). (D-E″) Targeted expression of *atoh7* using the *rx2*::Lex^PR^ *OP*::Lyn-Tomato *OP*::*atoh7, tp1-MmHbb*:d2GFP hatchlings results in upregulation of the Notch reporter and GFP expression (green) in Lyn-Tomato-positive MG cells (magenta) (*n*=8 fish, data obtained from two independent experiments). Scale bars: 20 µm.

To address whether Notch signaling is activated in the Atoh7-targeted MG cells, we generated a transgenic line using the *tp1-MmHbb*:d2GFP construct, successfully used in other systems as a bona fide Notch transcriptional reporter (Clark et al., 2012) (Fig. 4A). Upon the inhibition of Notch signaling by the γ-secretase inhibitor DAPT, reporter expression is strongly reduced throughout the animal, including the retina (Fig. S4A-B′). We observed Notch activity highlighted by GFP expression in *tp1-MmHbb*:d2GFP transgenic animals close to the CMZ, but never in quiescent MG cells (Fig. 4B-C‴). We then crossed *rx2*::Lex^PR^ *OP*::*atoh7 OP*::Lyn-Tomato to *tp1-MmHbb*:d2GFP medaka fish and proceeded with the induction schemes as previously described (Fig. 4D). We analyzed the retinae at 2 and 4 days after targeted *atoh7* induction and found that several MG cells expressed EGFP and therefore activated the Notch signaling pathway in response to *atoh7* expression (Fig. 4E-E″). The expression of the *tp1-MmHbb*:d2GFP reporter tightly correlates with the MG cells in which *atoh7* and Lyn-Tomato were induced (4 days: 71%, *n*=613 cells; 2 days: 31%, *n*=140 cells). These data demonstrate that targeted induction of *atoh7* in quiescent MG cells of the fish retina activates Notch signaling.

### Induction of NICD is sufficient to trigger MG cell proliferation

**Fig. 5.**
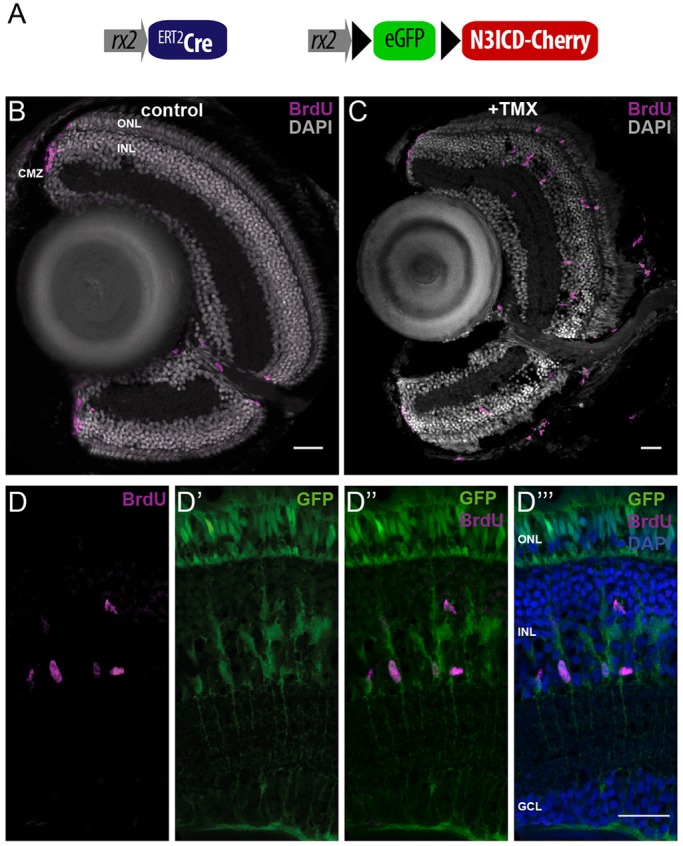
**NICD overexpression induces MG cells to re-enter the cell cycle.** (A-D‴) Targeted expression of the N3ICD drives MG cells into S phase. BrdU incorporation was assessed in controls (B) or tamoxifen-induced (+TMX) *rx2*::LoxP/EGFP/LoxP/N3ICD-Cherry hatchlings (C). BrdU incorporation (magenta) was detected in the CMZ (B) but not in the differentiated part of control retinae (*n*=4 fish, data obtained from two independent experiments). Targeted NICD expression results in BrdU incorporation in MG cells and photoreceptors in the central retina (C-D‴), colocalizing with EGFP expression (*n*=4 fish, data obtained from two independent experiments). Scale bars: 20 µm.

Our results suggested that the activation of MG cells by targeted expression of *atoh7* is mediated by the upregulation of Notch signaling. To test whether Notch activity is sufficient to trigger MG cell mitotic activity, we inducibly expressed the Notch intracellular domain (NICD) in MG cells via Cre/LoxP-mediated recombination using the *rx2*::*^LoxP^*N3ICD transgenic fish line (*rx2*::LoxP/EGFP/LoxP/N3ICD-Cherry) (Fig. 5A). Induction of N3ICD was achieved by triggering nuclear translocation of Cre recombinase via tamoxifen in double transgenic *rx2*::*^LoxP^*N3ICD and *rx2*::^ERT2^Cre fish (Reinhardt et al., 2015). We assayed at the same developmental stages as for *atoh7* expression in our previous experiments and incubated the fish in BrdU for 3 days post induction. Upon N3ICD expression, we detected a massive accumulation of BrdU-positive MG cells in comparison with non-induced controls [induced fish (*n*=4): 145 BrdU-positive MG per fish; non-induced control fish (*n*=4): 0 BrdU-positive MG] (Fig. 5B-D‴). This result shows that induction of constitutively active N3ICD is sufficient to stimulate cell cycle re-entry of MG cells.

### Induced Atoh7 favors the formation of neurogenic clusters

**Fig. 6.**
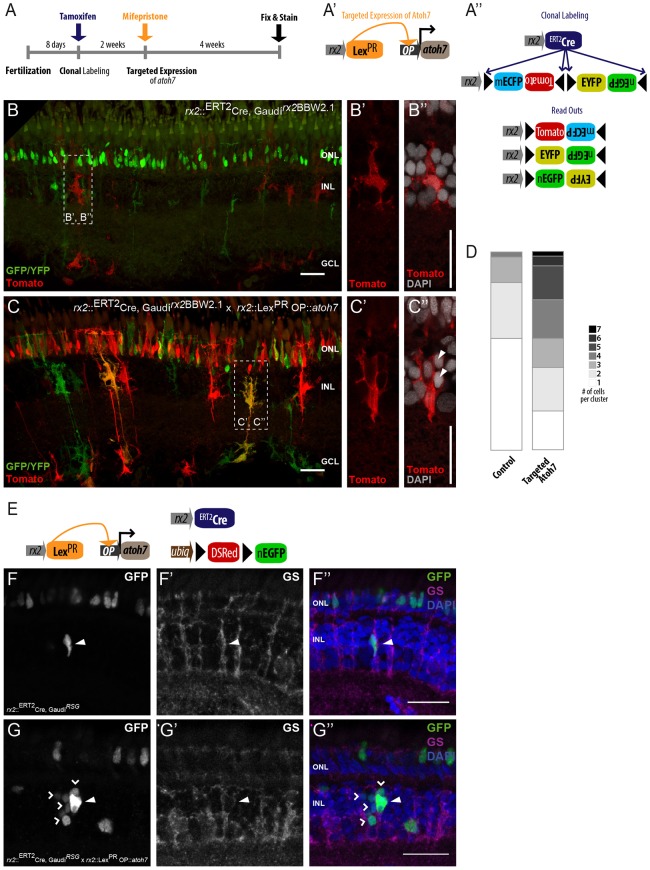
**Targeted *atoh7* drives** **neurogenic cluster formation of** **clonal MG cells.** (A-A″) Induction scheme (A) and constructs used for targeted induction of *atoh7* (A′) and clonal labeling (A″). The *rx2*::^ERT2^Cre transgenic line mediates excision or inversion events in the Gaudí*^rx2^*^BBW2.1^ cassette that result in three possible FP readouts (A″), which will be expressed by daughter cells that maintain the MG cell fate. (B-C″) In the central retina, recombination is targeted to MG cells and photoreceptors (B,C). In control retinae, MG cells display a compact nucleus and processes spanning from the apical to the basal domains of the neural retina (*n*=39 clones from three fish, data obtained from two independent experiments) (B′,B″). In *atoh7*-expressing fish, MG cells form clusters containing several nuclei (arrowheads) (*n*=41 clones, from four fish, data obtained from two independent experiments) (C-C″). (D) Quantification of numbers of nuclei per cluster shows that targeted induction of *atoh7* results in clusters containing more nuclei than those of controls. (E) Constructs used for targeted induction of *atoh7* and nuclear clonal labeling. The *rx2*::^ERT2^Cre transgenic line mediates excision of DSRed resulting in nuclear EGFP expression. (F-F″) Without induction of *atoh7*, single nuclear EGFP (white/green)-labeled GS-positive cells (white/magenta) (arrowhead) are present in the INL. (G-G″) Induction of *atoh7* induces the formation of nuclear EGFP (white/green)-labeled clusters (open arrowheads). One nucleus (arrowhead) is positive for GS (white/magenta) (*n*=6 fish, data obtained from two independent experiments). Scale bars: 20 µm.

To investigate the expansion and the lineage of the re-activated MG cells, we used the Gaudí toolkit, which allows multicolor labeling of progenitors, stem cells and their descendants via Cre/LoxP mediated recombination (Fig. 6A-A″) (Centanin et al., 2014; Livet et al., 2007). To follow the expansion of MG cells, we induced stochastic and sparse recombination by a mild tamoxifen induction (see scheme of treatment in Fig. 6A) of the *rx2*::^ERT2^Cre line in the background of an *rx2-*driven Gaudí*^rx2^*^BBW2.1^ recombination reporter (Fig. 6A″). This approach labels individual MG cells and those descendants that maintained Rx2 expression. After a chase of 4 weeks, we observed predominantly single cells and clusters of two cells among the labeled MG cells of control retinae (Fig. 6B,D). By contrast, when clonal labeling was combined with the triggering of *atoh7* expression, the majority of MG cells formed clonal clusters of three or more nuclei (Fig. 6C,D). To achieve exclusively nuclear labeling, we used *rx2*::^ERT2^Gaudí^RSG^ fish in combination with *atoh7* inductions to analyze cluster formation (Fig. 6E). Supporting the findings of the previous experiment, control fish displayed single nuclear EGFP-labeled MG cells (Fig. 6F-F″), whereas upon targeted induction of *atoh7* the formation of multicellular neurogenic clusters is triggered, as highlighted by nuclear-tagged EGFP (Fig. 6G-G″). These data demonstrate that the targeted induction of *atoh7* in MG cells triggers the formation of neurogenic clusters highly reminiscent of the neurogenic clusters formed by zebrafish MG cells in response to intense light treatment or mechanical injuries (Fausett and Goldman, 2006; Kassen et al., 2007; Thummel et al., 2008).

### MG cells produce neurons in response to targeted *atoh7* expression

**Fig. 7.**
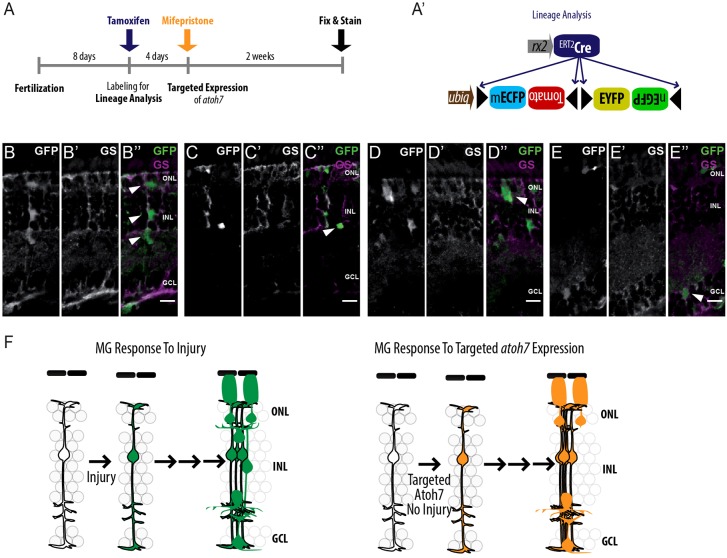
**Neural differentiation of MG cells upon targeted expression of *atoh7*.** (A,A′) Induction scheme (A) and constructs used for lineage analysis (A′). The *rx2*::^ERT2^Cre transgenic line mediates excision or inversion events in the Gaudí^BBW2.1^ cassette that result in three possible FP readouts (see Fig. 5A″), which will be expressed by all daughter cells irrespective of their fate. (B-E″) Lineage of MG cells upon targeted *atoh7* expression (*n*=3 out of six fish, data obtained from three independent experiments). Recombined EGFP-positive nuclei (white/green) located on one GS-positive MG process (white/magenta) can be found in the INL, the inner plexiform layer and the ONL (B″, arrowheads). Clusters of EGFP-positive cells are found in the ONL (D″, arrowhead). Single EGFP-positive cells can be detected in the amacrine cell layer (C″, arrowhead) and the RGC layer (E″, arrowhead). Scale bars: 10 µm. (F) MG cells respond to injuries by upregulating various transcription factors, which leads to proliferation, differentiation and regeneration of the lost cell types. Upon targeted induction of *atoh7* in MG cells, proliferation and differentiation are induced even in the absence of an injury.

The full differentiation potential of an induced MG cell can only be addressed by following its entire lineage. We achieved that by using the ubiquitous Gaudí*^BBW2.1^* transgenic line, which allows labeling of cells within a lineage irrespective of their fate (see scheme in Fig. 7A,A′). We triggered recombination in *rx2*::^ERT2^Cre Gaudí*^BBW2.1^* control fish to follow the lineage of individual MG cells during homeostasis. We allowed the lineage to progress for 2 weeks and analyzed clones expressing nuclear EGFP, because it is the only fluorophore that labels nuclei unambiguously. In control fish, we observed labeled cells in the central retina only within the *rx2* expression domain, i.e. MG cells and photoreceptors (data not shown). By contrast, when clonal labeling was followed by targeted *atoh7* expression we found nuclear-labeled cells representing a clonal lineage distributed in all three nuclear layers (Fig. 7B-E″). Clonal derivatives of MG cells were negative for GS staining and exhibited the typical morphology of photoreceptor progenitors, amacrine cells and RGCs (Fig. 7B-E″). Together, these data show that a transient *atoh7* induction in MG cells within a differentiated retina is sufficient to trigger a regeneration-like response that includes re-entry into the cell cycle and *de novo* neurogenesis *in vivo* (Fig. 7F).

## DISCUSSION

Our data demonstrate that the targeted expression of a single factor, Atoh7, in MG cells recapitulates the regeneration response in the uninjured fish retina. The response includes cell cycle re-entry, formation of neurogenic clusters and differentiation of clones into different cell types. We obtained these results by establishing an inducible transactivation system combined with long-term lineage analysis.

Atonal genes are well-known regulators of neurogenesis during organ formation (Brown et al., 1998; Jarman et al., 1994; Livesey and Cepko, 2001). Until now, Atoh7 was considered to be a transcription factor that channels proliferating cells into differentiation. Additionally, *atoh7* was reported to be expressed during the neurogenic phase that follows injury of the zebrafish retina (Fimbel et al., 2007; Sherpa et al., 2008). Here, we uncover an unexpected, new expression of *atoh7* in uncommitted, proliferating progenitors during retinal homeostasis and regeneration. The expression of *atoh7* directly adjacent to the Rx2-positive stem cells of the CMZ is transient, hinting at a dual function of Atoh7, initially in proliferation and subsequently in terminal differentiation of retinal progenitor cells.

We show that Atoh7 is sufficient for driving quiescent MG cells back into the cell cycle. We have previously reported several cell cycle regulators as downstream targets of Atoh7 (Del Bene et al., 2007). Many of these genes have been linked initially to cell cycle exit during early retinogenesis. This assumption needs to be re-evaluated in the light of Atoh7-induced cell cycle re-entry of MG cells. One target that is particularly interesting in this context is Alcama. It has been reported recently that Alcama is a novel marker of retinal neuroepithelial cells and its expression was shown to be upregulated after injury in proliferating MG cells (Nagashima et al., 2013). It is possible that the expression of Alcama in these cells is regulated by Atoh7 during both retinal homeostasis and regeneration.

In Atoh7 induction paradigms, we found that BrdU incorporation in Atoh7-induced MG cells also occurred to some extent in GFP-negative MG cells. This could be due to the binding of the LexPR transcription factor to only one of the two OP sites present in a cell. Our experiments have shown that ∼90% of cells co-express both OP sites, and the remaining 10% express either one or the other. Another reason could be a non-cell-autonomous action of Atoh7. It has been shown previously in zebrafish that injury-activated MG cells produce and respond to secreted signaling molecules (Wan et al., 2014; Zhao et al., 2014). In the case of Atoh7 induction, MG cells could also start to express and secrete such molecules to activate adjacent MG.

A central factor during retinal regeneration in zebrafish is the proneural transcription factor Ascl1a, which is a key regulator of MG cell activation after injury (Fausett et al., 2008). Ascl1a has been reported to activate expression of *lin-28* as well as Notch signaling (Ramachandran et al., 2010; Wan et al., 2012). Interestingly, we have previously reported Lin-28 as a downstream target of Atoh7 (Del Bene et al., 2007). Furthermore, our results in this study show that Atoh7 induction in MG cells activates Notch signaling, placing Atoh7 parallel to or downstream of Ascl1a. Strikingly, except Atoh7, none of several additional factors (including Ascl1a) had the potential to trigger proliferation and differentiation when tested in the system described here (Table S1).

Our results position Notch signaling downstream of Atoh7 during the regeneration-like response of MG cells, although it does not immediately respond to injury (data not shown). Strikingly, Notch activation by N3ICD expression is also sufficient to trigger MG proliferation. When we analyzed the lineage of N3ICD-expressing MG cells, we found, as in the case of Atoh7, differentiation into neurons, preferentially RGCs (Fig. S5A-D′). The role of Notch in the regenerating vertebrate retina has been previously reported both in chicken and in rodents (Del Debbio et al., 2010; Ghai et al., 2010; Hayes et al., 2007), where activation of the pathway leads to increased proliferation of MG-derived progenitors and Notch inhibition prevents MG proliferation. These were contrasted by the findings in zebrafish, where inhibition of Notch signaling induces MG cell proliferation in the absence of injury (Conner et al., 2014). Interestingly, our results indicate that medaka is highly reminiscent of higher vertebrates regarding the role of Notch in inducing proliferation of MG cells, and expand the previous roles reported for Atoh7. The conservation of Atonal genes leads to the question of whether its new role is maintained across the vertebrate lineage, which would position Atonal-related genes as crucial targets for regenerative approaches.

## MATERIALS AND METHODS

### Animals and transgenic lines

Medaka (*Oryzias latipes*) fish used in this study were kept as closed stocks in accordance to Tierschutzgesetz 111, Abs. 1, Nr. 1 and with European Union animal welfare guidelines. Fish were maintained in a constant recirculating system at 28°C on a 14 h light/10 h dark cycle (Tierschutzgesetz 111, Abs. 1, Nr. 1, Haltungserlaubnis AZ35–9185.64 and AZ35–9185.64/BH KIT). The following stocks and transgenic lines were used: wild-type Cabs, *atoh7*::EGFP (Del Bene et al., 2007), *rx2*::H2B-RFP (Inoue and Wittbrodt, 2011), Gaudí^BBW2.1^, Gaudí*^RSG^* (Centanin et al., 2014), *rx2*::^ERT2^Cre, Gaudí*^rx2^*^BBW2.1^, *cmlc2*::ECFP *OP*::EGFP, *rx2*::Lex^PR^ *OP*::EGFP, *rx2*::Lex^PR^ *OP*::*atoh7*, *rx2*::Lex^PR^ *OP*::*atoh7 OP*::Lyn-Tomato, *tp1-MmHbb*:d2GFP (Clark et al., 2012) and *rx2*::*^LoxP^*N3ICD. All transgenic lines were created by microinjection with Meganuclease (I-SceI) in medaka embryos at the one-cell stage, as previously described (Thermes et al., 2002), except for *tp1-MmHbb*:d2GFP which was created by microinjection with Tol2. See Table S2 for sequences of the vectors used.

### Cloning of *atoh7*

The *O. latipes atoh7* cDNA was obtained from an expression library carried out previously in our laboratory. *Atoh7* was cloned into an I-SceI vector containing the *rx2* promoter (Martinez-Morales et al., 2009) and the Lex^PR^ *OP* cassette from Emelyanov and colleagues (Emelyanov and Parinov, 2008).

### Induction of the Lex^PR^ System, the Cre/lox system, BrdU incorporation and DAPT treatment

For induction of the Lex^PR^ System, hatchlings (stage 40) were induced by incubating them in a 2.5 to 5 µM mifepristone (M8046, Sigma-Aldrich) solution in embryo rearing medium (ERM; 17 mM sodium chloride, 0.4 mM potassium chloride, 0.27 mM calcium chloride dihydrate, 0.66 mM magnesium sulfate heptahydrate, pH7).

For induction of the *rx2*::^ERT2^Cre, hatchlings were treated with a 5 µM tamoxifen (T5648, Sigma-Aldrich) solution in ERM for 15 h and washed afterwards with ERM.

For BrdU incorporation, hatchlings were incubated in 1.6-2.5 mM BrdU (BB5002, Sigma-Aldrich) diluted in ERM.

For DAPT treatment, hatchlings were incubated in 50 μM DAPT diluted in ERM.

### Retinal injuries

Embryos at hatching stage were anesthetized in 1×Tricaine (A5040, Sigma-Aldrich). Under microscopic visualization, the right retina was stabbed multiple times in the dorsal part with a glass needle (0.1 mm diameter). Left retinae were used as controls.

### Genotyping for *OP*::*atoh7*

Fin clip tissue of treated fish from a cross of the *rx2*::LexPR *OP*::*atoh7 OP*::GFP line to the *rx2*::^ERT2^Cre, Gaudi*^rx2^* line were digested overnight at 37°C in Ten9 buffer (100 mM Tris-HCl pH 8.5, 10 mM EDTA, 200 mM NaCl, 1% SDS) with 0.9 mg/ml Proteinase K (Roche). The DNA was subsequently purified by phenol chloroform isoamyl alcohol (PCI) extraction. A standard genotyping PCR was performed using a forward primer binding the operator sequence and a reverse primer binding *atoh7* (forward primer: GAATCCTGTTGCCGGTCTTGCGATG; reverse primer: GACAGCTTTTTGTCTTGGCCCCACT).

### Detection of antigens and mRNA

Fluorescence whole-mount *in situ* hybridization was essentially carried out as described previously (Souren et al., 2009). To determine the identity of *atoh7*-expressing cells in the INL, an anti-GS stain was performed in combination with the fluorescence *atoh7 in situ* as described by Inoue and Wittbrodt (2011). For immunohistochemistry, embryos were fixed overnight in 4% paraformaldehyde (PFA) in PTW at 4°C and mounted for cryosectioning. Antibody staining was performed as described by Inoue and Wittbrodt (2011), using the following primary antibodies (1:500): anti-GS (mouse; Chemicon, MAB302), anti-EGFP (chicken; Life Technologies, A10262), anti-DsRed (rabbit; Clontech, 632496), rabbit anti-Rx2 (Reinhardt et al., 2015), anti-PCNA (mouse; Santa Cruz, sc-56) and anti-BrdU (rat; AbD Serotec, BU1/75). The following secondary antibodies were used (1:500): anti-mouse Cy5 (Jackson ImmunoResearch, 715-175-151), anti-chicken 488 (Jackson ImmunoResearch, 703-485-155), anti-rat DyLight549 (Jackson ImmunoResearch, 112-505-143), anti-rabbit DyLight549 (Jackson ImmunoResearch), anti-mouse Alexa546 (Life Technologies, A-11030) and anti-rat Alexa633 (Life Technologies, A21094). DAPI (Sigma-Aldrich, D9564) nuclear counterstaining was performed as described by Inoue and Wittbrodt (2011).

### BrdU antibody staining

BrdU antibody staining was performed with an antigen retrieval step. After all antibody stainings except for BrdU and DAPI staining was complete a fixation for 30 min was performed with 4% PFA. Slides were incubated for 2 h at room temperature in 2 N HCL solution, and pH was recovered by washing with a saturated Borax solution before incubation with the primary BrdU antibody.

### Imaging and image processing

All images were acquired by confocal microscopy (Leica TCS SPE and Leica SP5). Images were acquired with either 20× water objective or 40× oil objective. Images were processed using Fiji image processing software to adjust brightness and contrast, stitched (http://fly.mpi-cbg.de/~preibisch/software.html) if necessary, followed by application of the pure denoise plugin on the final picture with standard automated settings and six cycles of denoising (http://bigwww.epfl.ch/algorithms/denoise/).
